# Small bowel intussusception in an adult secondary to gastrointestinal stromal tumor: a rare case report

**DOI:** 10.1097/MS9.0000000000000458

**Published:** 2023-04-07

**Authors:** Sunil Basukala, Sabin Karki, Suman Maharjan, Sabin Banmala, Melina Shrestha, Mukesh Jayswal, Kala Shrestha

**Affiliations:** a Department of Surgery, Shree Birendra Hospital, Chhauni; b Nepalese Army Institute of Health Sciences – College of Medicine, Sanobharyang, Kathmandu, Nepal

**Keywords:** adult intussusception, case report, gastrointestinal stromal tumor, intussusception, small bowel intussusception

## Abstract

Intussusception is a rare surgical condition in adults less commonly confined to small bowel. Adult intussusception is an indication of surgical resection due to possibility of ischemia and malignant pathological cause like gastrointestinal stromal tumor (GIST) as in this case.

**Case presentation:**

A 32-year-old male presented with abdominal pain and vomiting for 3 days. Vitals parameters and abdominal examinations were normal. Abdominal ultrasonography revealed target sign suggesting ileoileal intussusception in right lower quadrant. Abdominal contrast-enhanced computed tomography of abdomen showed features suggestive of ileoileal intussusception. Diagnostic laparoscopy was done which was later turned to laparotomy for segmental resection and anastomosis of ileum for ileoileal intussusception. Polypoidal growth noted in the resected section of ileum was found to be GIST (CD117 and DOG-1 positive) which was considered to be the lead point. Patient recovered well during postoperative period and later referred to oncology clinic for chemotherapy.

**Clinical discussion:**

Intussusception and subsequent obstruction are very uncommon presentation in a patient with GIST because of their tendency to grow in an extraluminal fashion. As intussusception is rare in adult, high level of suspicion and proper imaging technique plays important role in diagnosing the condition.

**Conclusion:**

Ileoileal intussusceptions due to GIST are a rare clinical entity in adult intussusceptions and generally have a vague variable clinical presentation thus requiring high index of clinical judgement and suspicion with judicious use of imaging.

## Introduction

Highlights

- Intussusception refers to telescoping of proximal segment into lumen of distal segment of bowel causing obstruction, ischemic injury, and death of affected segment.
- Intussusceptions are uncommon in adults with only about 5% of all the cases among adults and 0.003–0.02% of adult hospital admissions. Approximately 90% of the intussusceptions in adult have a pathological lesion as lead point in which 65% of them are considered to be benign or malignant neoplasms including gastrointestinal stromal tumor (GIST).
- GISTs are rare clinical conditions, representing less than 0.2% of all gastrointestinal (GI) tumors and only 0.04% of small intestine malignant neoplasms which originate from interstitial cells of Cajal.
- Diagnosing intussusception in an adult is tough due to absence of classical symptoms, varying presentation, less dramatic and resembling various other disorders of GI tract. Computed tomography (CT) provides a confident identification of intussusception.
- Surgical resection is the definitive treatment of GIST with adjuvant treatment with imatinib.

Intussusception refers to telescoping of proximal segment into lumen of distal segment of bowel causing obstruction, ischemic injury and death of affected segment^1^. Such condition is an indication for surgery due to possibility of ischemia and malignant lead point in adults^2^. Intussusception generally occurs in fifth and ninth months of the life and are uncommon in adults with only about 5% of all the cases among adults and 0.003–0.02% of adult hospital admissions^1^,^3^,^4^. GISTs are rare clinical conditions, representing less than 0.2% of all GI tumors and only 0.04% of small intestine malignant neoplasms which originate from interstitial cells of Cajal and can occur as a lead point in intussusceptions of adults^5^. They may occur anywhere along the GI tract with 60–70% occurring in the stomach and 20–25% in the small bowel^6^. Intussusception confined to small bowel is rare and accounts for about 10% of all the intussusceptions in adults with few of the studies showing as low as 1%^3^. The diagnosis of the condition is simple with palpable abdominal mass in most of the cases (>50%)^3^. Intussusception in adult are generally located in ileocolic part or colonic intussusception^7^. Intussusception also presents with vomiting along with abdominal pain, however, in ileoileal intussusceptions the presentation may be vague leading to delay in diagnosis resulting intraoperative diagnosis^3^. Intussusception requires proper imaging modalities for the diagnosis and warrants definitive surgical management.

Herein, we report a rare case of ileoileal intussusceptions that was managed successfully with segmental resection and anastomosis which later found to be caused due to GIST as a lead point. This case report has been reported in line with the SCARE 2020 criteria^8^.

## Method

We reported this case following the updated consensus-based Surgical Case Report (SCARE) Guidelines^8^.

## Case presentation

A 32-year-old male, serving soldier, with no known comorbidities was referred to our tertiary center with complain of abdominal pain and vomiting for last 3 days on the day of presentation. The pain was sudden in onset, spasmodic in nature, intermittent in type, localized to right lumber and periumbilical region, nonradiating, associated with nausea and abdominal fullness, aggravated by eating food, and no known relieving factors. He also gave history of two episodes of nonbilious and nonblood stained vomiting. There was no history of any trauma, fever, hematemesis, tenesmus, hemoptysis, significant weight loss, hematochezia, abdominal tenderness, night sweat, diarrhea, cough, icterus, and chest pain.

He had no history of similar illness in the past. His past history, family history and allergic history were unremarkable. He was nonsmoker, did not consume alcohol and had normal bowel and bladder habit.

On examination his vitals parameters were stable and the patient looked fair without abdominal tenderness or distention and no palpable abdominal mass. The laboratory analyses were sent and it showed decrease in platelets count of 134 000 cells/cumm with normal level of hemoglobin of 12.6 g/dl and total leukocyte count of 10 800 cells/cumm with normal range of other laboratory parameters, serology was nonreactive for HIV, hepatitis B, and hepatitis C.

Ultrasonography of abdomen was performed which showed bowel in bowel sign in the right lower quadrant measuring 4×4 cm extending over 8 cm giving target sign likely suggestive of ileoileal intussusceptions. Also, contrast-enhanced CT of abdomen and pelvis revealed features suggestive of ileoileal intussusception with preserved vascularity and with no features of bowel obstruction. Along with this colonoscopy was done and showed ileal ulcers and biopsy was taken in the same setting.

**Figure 1:**
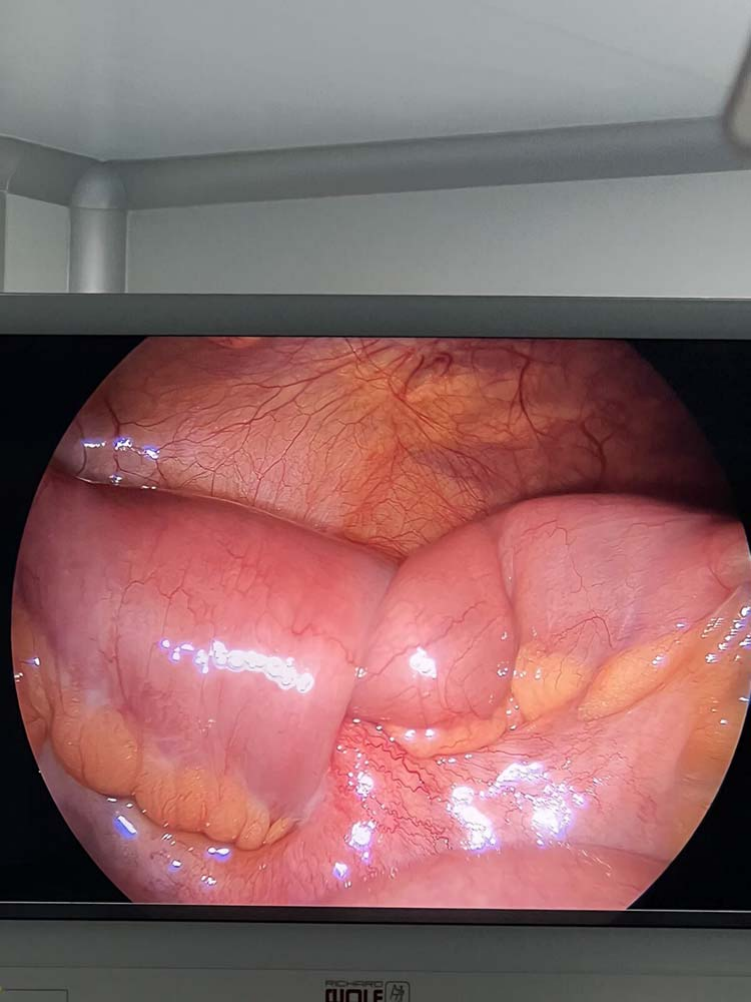
Ileoileal intussusception in the distal ileum.

**Figure 2:**
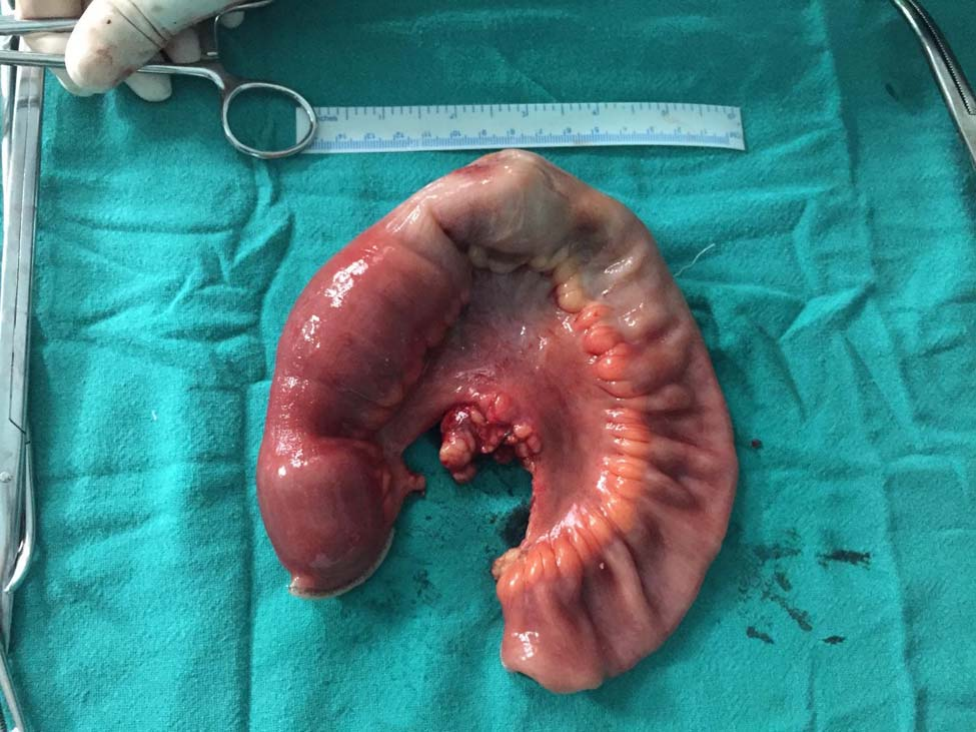
The resected segment of ileum.

**Figure 3:**
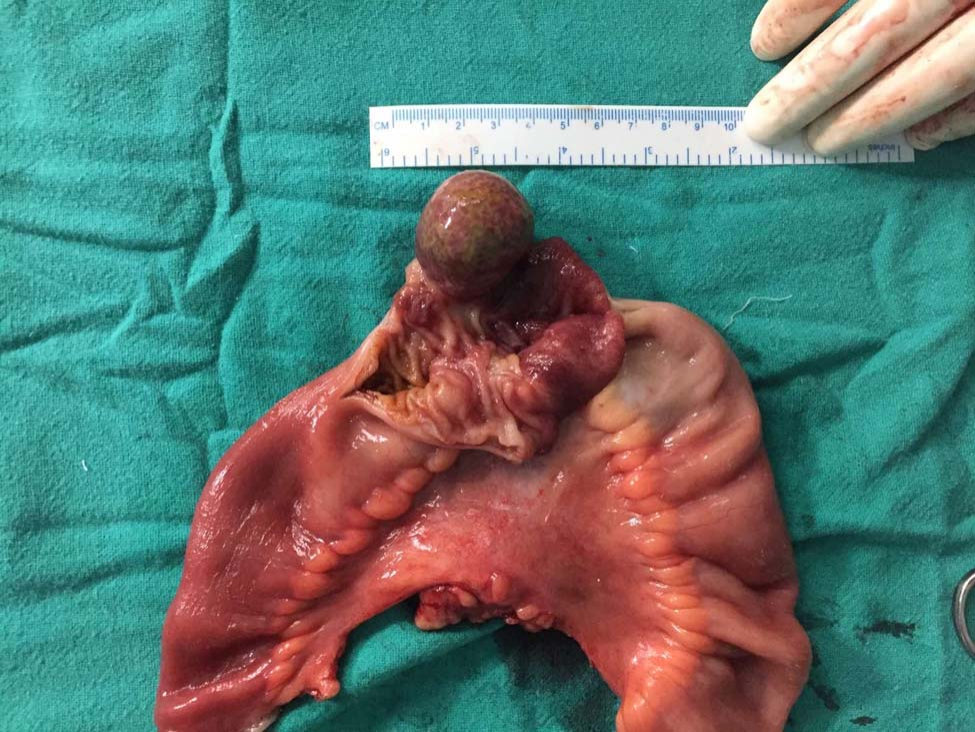
A polyp as a lead point of intussusceptions in the resected segment of ileum.

With adequate evidence and confirmation from clinical presentation of patients and investigatory findings suggesting ileoileal intussusception, the decision for need of surgery was made. The patient underwent diagnostic laparoscopy (Fig. 1) along with laparotomy for segmental resection and anastomosis of ileum for ileoileal intussusception. Intraoperatively, ileoileal intussusception in the distal ileum which was 15 cm from I-C junction was found as shown in Figure 1. The resected segment of ileum on macroscopic examination showed dark brown soft tissue mass (Fig. 2). Along with the discovery of intussusceptions, a polypoidal growth in the ileum was noticed which was considered to be the lead point (Fig. 3). Mesenteric lymph nodes were not enlarged; thus, lymph node excision was not done.

**Figure 4:**
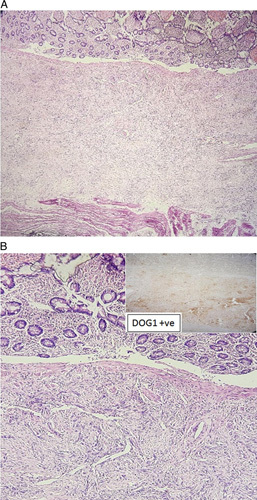
(A) Submucosal layer shows spindle cells as well as epithelioid cells in diffuse sheets and nests. (B) Spindle cells in syncytial clusters with elongated nuclei and inconspicuous nucleoli. immunohistochemistry with DOG-1 shows nuclear positivity.

Histopathological examination of the specimen turned out to be GIST, which was positive for CD117 and DOG-1 in immunohistochemistry (Figs. 4A, B). Patient recovered well during postoperative period and was discharged on 10th postoperative day. Patient was then referred to oncology clinic for chemotherapy and was started on imatinib 400 mg as an adjuvant therapy. Patient was asymptomatic and stable during his scheduled outpatient follow-up 1 month after the discharge.

## Discussion

Adult intussusceptions are uncommon and rarely diagnosed preoperatively^9^,^10^. Intussusception has equal prevalence in both sexes in adults^11^. The typical presentation being abdominal pain, palpable mass, vomiting, and bloody/currant jelly stools can be observed in pediatric ages, however, diagnosing intussusception in an adult is tough due to absence of classical symptoms, varying presentation, less dramatic, and resembling various other disorders of GI tract^1^,^7^. Similar variable clinical presentations are reported in many other case reports of intussusceptions due to GIST^6^,^12^–^14^.

Abdominal ultrasonography has high sensitivity and specificity in diagnosing intussusceptions, while abdominal radiographs are important to rule out any obstruction in emergency setting^11^,^15^. CT provides a confident identification of intussusception and is superior than other studies; ultrasonography and endoscopy in patient with nonspecific abdominal complaints^4^,^16^. Few other investigations like barium enema, colonoscopy or sigmoidoscopy or upper GI series can be used judiciously according to the need^17^.

Unlike intussusception in children where 90% of cases are idiopathic, ∼90% of the intussusceptions in adult have a pathological lesion as lead point in which 65% of them are considered to be benign or malignant neoplasms including GIST^4^,^9^. Colon is the more likely site of malignancy than small bowel. Less common causes of intussusception in adult include postoperative factors (adhesions, suture lines), polyps, Meckel’s disease, sprue, cecal duplication, and intramural hematoma. Any intraluminal lesion, especially polyps, alter normal peristaltic activity producing an area of sequence of constriction and relaxation, thus telescoping the leading point through the distal bowel lumen^12^.

GISTs are pathologically defined by positive immunostaining for c-kit proto-oncogene – CD117 (overexpressed in 95%) and CD34 (positive in 60–70%) (other possible markers include DOG-1, desmin, vimentin, and others)^18^. GISTs have highly variable clinical presentation depending upon their size, location, and presence of mucosal ulceration. Abdominal pain and GI bleeding are the most common symptoms or signs, while intussusception and subsequent obstruction are very uncommon presentation because of their tendency to grow in an extraluminal fashion^5^. Our case presented with intussusception which is probably caused due to stromal tumor in the ileum.

Treatment of adult intussusception is always surgical. However, management of intussusceptions remains debatable versus primary *en bloc* or initial reduction followed by more limited resection^4^. There still remains a challenge is differentiating malignancy from benign etiology in preoperative and intraoperative timing thus possessing danger of transperitoneal seeding, venous embolization, perforation during manipulation and handling of friable malignant tissue advising against primary and intraoperative reduction of colonic intussusceptions (54–77% malignancy rates in colonic intussusceptions) or intussusceptions in age greater than 60, however, small bowel intussusceptions with lesser occurrence of malignancy can be gently treated with primary reduction and small resection^4^,^9^,^19^. Intraoperative histopathology can help determining the extent of surgical resection in advance facilities^11^. Thus, resection is the treatment of choice in adults with intussusceptions without any trial of manual reduction^20^.

The treatment of choice for localized GIST is complete surgical resection. Routine removal of lymph nodes is typically not necessary as lymph node metastases are rare. Adjuvant treatment with imatinib, tyrosine kinase inhibitor following surgical resection of GISTs can significantly reduce the risk of disease recurrence^6^,^12^,^18^.

## Conclusions

Ileoileal intussusception due to GIST is a rare clinical entity in adult intussusception. Its diagnosis is quite challenging and difficult because of the rarity of the condition itself and the nonspecific presentation. CT represents the definitive diagnositic imaging modality comparative to other imaging modalities. And surgical resection is the definitive treatment of ileoileal intussusceptions GIST. Adult intussusceptions and GIST generally have a vague variable clinical presentation thus requiring high index of clinical judgement and suspicion with judicious use of imaging.

## Ethical approval

None.

## Consent

Written informed consent was taken for the publication of the case report and the images. A copy of it is available for review by editor in chief of this journal on request.

## Sources of funding

None.

## Author contribution

S. Basukala, S.K., S.M., S. Banmala, M.S., M.J., and K.S. were involved in writing, editing, and review of the manuscript. All the authors approved the final version of the manuscript and agreed to be accountable for all aspects of the work.

## Conflicts of interest disclosure

The authors declare that they have no financial conflict of interest with regard to the content of this report.

## Research registration unique identifying number (UIN)

None.

## Guarantor

Sabin Karki.

## Data availability statement

Not applicable.

## Provenance and peer review

Not commissioned, externally peer reviewed.
